# Modeling of Structure H Carbon Dioxide Clathrate Hydrates: Guest–Lattice Energies, Crystal Structure, and Pressure Dependencies

**DOI:** 10.1021/acs.jpcc.2c04140

**Published:** 2022-08-26

**Authors:** Adriana Cabrera-Ramírez, Rita Prosmiti

**Affiliations:** † Institute of Fundamental Physics (IFF-CSIC), CSIC, Serrano 123, 28006, Madrid, Spain; ‡ Doctoral Programme in Theoretical Chemistry and Computational Modelling, Doctoral School, Universidad Autónoma de Madrid, 28049, Madrid, Spain

## Abstract

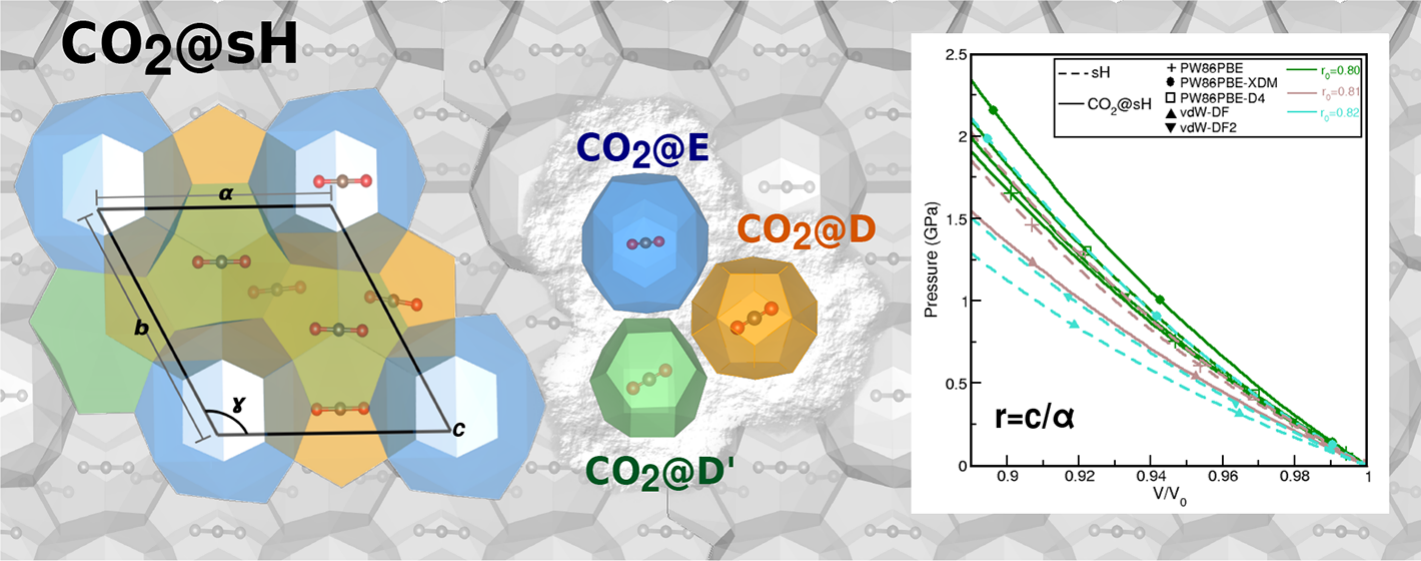

We performed first-principles computations to investigate the complex interplay of molecular interaction energies in determining the lattice structure and stability of CO_2_@sH clathrate hydrates. Density functional theory computations using periodic boundary conditions were employed to characterize energetics and the key structural properties of the sH clathrate crystal under pressure, such as equilibrium lattice volume and bulk modulus. The performance of exchange–correlation functionals together with recently developed dispersion-corrected schemes was evaluated in describing interactions in both short-range and long-range regions of the potential. Structural relaxations of the fully CO_2_-filled and empty sH unit cells yield crystal structure and lattice energies, while their compressibility parameters were derived by including the pressure dependencies. The present quantum chemistry computations suggest anisotropy in the compressibility of the sH clathrate hydrates, with the crystal being less compressible along the *a*-axis direction than along the *c*-axis one, in distinction from nearly isotropic sI and sII structures. The detailed results presented here give insight into the complex nature of the underlying guest–host interactions, checking earlier assumptions, providing critical tests, and improving estimates. Such entries may eventually lead to better predictions of thermodynamic properties and formation conditions, with a direct impact on emerging hydrate-based technologies.

## Introduction

Clathrate hydrates are ice-like crystalline solids that are constituted of a network of interlinked hydrogen-bonded water molecules in the form of polyhedral cages (host lattice), where gas molecules are encapsulated as guests. In nature, they have been observed in the form of natural gas hydrate deposits on the ocean floor and in the Earth’s permafrost regions, as well as in other planetary bodies inside and outside the solar system.^1−11^ There are three common types of gas hydrate structures, including cubic sI and sII and hexagonal sH, which depend on the size and type of cages and their connectivity. The properties of the guest molecule(s), such as shape, size, and type, determine the variant of the clathrate hydrate structure formed under specific thermodynamic conditions. The van der Waals (vdW) forces maintain the stability of clathrate at the appropriate temperature and pressure, and their stability relies on the nature of the guest molecule and its interactions with the hydrogen-bonded water network.

Research in gas hydrates is in constant development due to new challenges related to their properties and potential applications in the energy industry, environment and climate, and materials design. Thus, investigations in the field have been focused on new energy supply sources, such as methane and hydrogen clathrates,^12−17^ while mixed-binary clathrates, such as those containing CO_2_ and CH_4_ as guest gases, have been proposed as possible byproduct storage media and fuel sources.^15,18−20^ Recent advances have suggested that the capture and storage of greenhouse gases are of outstanding importance and a major challenge for gas-control technologies.^1,12,21,22^

Carbon dioxide clathrate hydrates present an excellent source for gas storage, and thus they are of potential use in CO_2_ sequestration.^23,24^ In virtually all these applications, gas hydrate properties, such as formation, stability, occupancy capacity, compressibility, and thermal expansion, are of critical importance, and theoretical insights into the underlying factors that determine them are valuable, especially when considering environmental and energy supply applications. Understanding fundamental properties of these gas hydrates allows the comprehension of the thermodynamic conditions and the dynamics that propitiate the CO_2_ release as well as the determination of the physicochemical processes involved. Even despite the CO_2_ capture prospects, the molecular level interactions between guest and host are generally lacking or still not well-characterized in the literature. In general, semiempirical models have been used to get a microscopic understanding of the underlying guest–host interactions as well as their effect on macroscopic properties of the gas hydrates.

From a theoretical perspective, an accurate description of the underlying interactions represents a challenging computational task, as both intramolecular covalent and hydrogen bonds of the host water network, as well as the intermolecular noncovalent interactions between the guest molecules and host lattice, should be represented at the same level of theory. Although wave function based (WF) and density functional theory (DFT) methods could describe guest–host molecular interactions in a reliable way with advantages in terms of their efficiency and computational performance,^25−33^ the validity of traditional DFT and modern dispersion-corrected DFT approximations^34−40^ in describing accurately both the hydrogen bond and dispersion interactions present in gas hydrates should be checked out.^25,41−46^ In this vein, the interaction of the CO_2_ molecule in isolated sI, sII, and sH clathrate cages has been investigated through different quantum chemistry methods,^25,44^ while current challenges involve investigations for the entire periodic clathrate hydrate unit cells via reliable approaches.^33,35,41,45−48^ Thus, a systematic evaluation of important aspects of the underlying interactions, such as cooperative guest–host and guest–guest/cage–cage effects should be performed, with the CO_2_@sH hydrate crystal properties being of interest, due to the high storage capacity expected compared to the corresponding sI or sII clathrates.^26,49^

On the other hand, experimental studies have focused on the synthesis of clathrate hydrates and on structural and spectroscopic measurements by means of X-ray and neutron diffraction, solid-state nuclear magnetic resonance (NMR), and Raman and infrared spectroscopic analysis with various guest molecules.^50−53^ To understand the effect of size, type, and flexibility of guest molecules on lattice structure sH and stability, X-ray diffraction measurements have been carried out.^54,55^ A majority of studies have concentrated on enclathration of the CO_2_ as a co-guest in the sH structure, such as those with CO_2_ + N_2_ gas mixtures, to explore cage-specific guest distributions and structural transitions in the process of CO_2_ capture and sequestration.^56^ Despite the above-mentioned motivations, there is no theoretical study for the CO_2_@sH clathrate, with most of the research focused on sH structures with methane (CH_4_), hydrogen (H_2_), nitrogen (N_2_), or mixed-binary sH clathrates,^54,57−68^ investigating thermodynamic stability,^69,70^ investigating the storage capacity of different hydrocarbon molecules,^62^ or predicting hydrate phase equilibrium and formation conditions^71^ or mechanical, acoustic, and thermal properties for sH hydrate structures using first-principles methods^72^ and combinations of approaches such as hybrid density functional theory and force-field methods.^73^

Following our recent studies on CO_2_ clathrates, our present study seeks to determine the stability of sH clathrates of CO_2_, as its storage capacity could make such clathrates good candidates for CO_2_ sequestration. CO_2_@sH clathrate has not been synthesized yet, and as in general the sH structure requires large molecules to occupy and stabilize its large E cage at low pressure, its stabilization could be also supported by the enclathration of clusters of two or more CO_2_ molecules.^26^ A total understanding of the properties of the sH hydrates is not yet complete, and in particular, the study of the behavior of CO_2_@sH hydrate is still under development. Therefore, we consider conducting the present research as a first attempt to represent interactions from first-principles methodologies toward investigation on multiple CO_2_ cage occupancies in such clathrate systems. This work aims at a better comprehension of the stability and description of the guest/host and host/host interactions in CO_2_@sH hydrate, by employing current state-of-the-art computational approaches seeking to improve our understanding of the complex behavior of periodic molecular crystals and to explore the factors playing a stabilizing role of immediate relevance in CO_2_ storage applications.

## Computational Details

**Figure 1:**
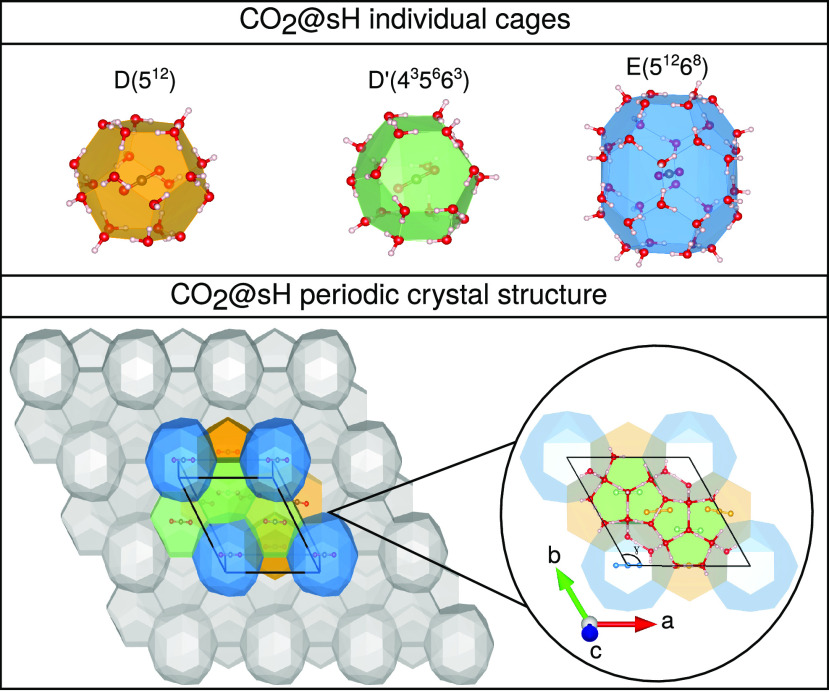
(upper panel) CO_2_@5^12^ (D), CO_2_@4^3^5^6^6^3^ (D′), and CO_2_@5^12^6^8^ (E) individual clathrate-like cages. (lower panel) Three-dimensional view of CO_2_@sH clathrate hydrate crystal structure. The box indicates the unit cell, while red, gray, and brown correspond to oxygen, hydrogen, and carbon atoms, respectively.

The CO_2_@sH clathrate hydrate crystal structure is shown in Figure 1 (lower panel) together with its CO_2_@sH unit cell, where a network of 34 water molecules forms a hexagonal structure of six cages of three different types (upper panel): three pentagonal dodecahedral 5^12^ or D cages, two irregular dodecahedral 4^3^5^6^6^3^ or D′ cages, and one icosahedral 5^12^6^8^ or E cage. The structure of water molecules shapes a hexagonal unit cell (space group *P*6/*mmm*) with three lattice parameters *a* = *b* and *c*, three angles α = β = 90° and γ = 120°, and volume *V* = (√3/2)*a*^2^*c*.

The initial crystallographic oxygen atom framework of the empty sH crystal was obtained from single crystal X-ray diffraction experiments,^74^ while the position and orientation of protons in water molecules were taken from refs (70 and 75). The proton arrangements have been determined according to simple ice rules with zero dipole moments in the unit cell.^70^ The dimensions of each side of the hexagonal unit cell are *a* = 12.212 Å and *c* = 10.143 Å, with the lowest dipole moment and potential energy proton-ordered form obtained from the TIP4P water model. The filled CO_2_@sH structure was generated by placed initially a single CO_2_ molecule at the center of all sH cages (see Figure 1).

For both filled CO_2_@sH and empty sH unit cells, electronic structure DFT calculations were performed with the use of the Quantum Espresso (QE)^76,77^ code. The plane-wave/pseudopotential approach within the projector-augmented-wave (PAW) method,^78^ as implemented in the QE code,^76,77^ was employed. All reported results used the standard PAW potentials supplied within QE, with PBE-based potentials as implemented in the LIBXC library.^79^ We also used the standard implementation in the LIBXC library^79^ for the PW86PBE functional. On the one hand, as semilocal DFT functionals are unable to describe dispersion forces that take place in intermolecular and intramolecular contexts, and whose effects need to be quantified, modern approaches account for long-range electron correlation; in particular, semiclassical treatments of the dispersion interaction are considered by DFT-D approaches. Such methods approximate the total molecular energy as a sum of mean-field energy, obtained in this work with the PW86PBE functional, and a dispersion energy contribution. The parameters considered in the different DFT-D approximations depend on the interatomic distance, the atomic dispersion coefficients, and damping functions to evaluate the dispersion energy between atom pairs.^36^ Here, we have considered the XDM and D4 dispersion correction schemes,^80−83^ which contain three-body intermolecular dispersion contributions. The XDM has been parametrized for the PW86PBE functional,^81^ and has been implemented in the QE package,^77^ while the D4 correction to the energy was included as a postprocessing using the DFTD4^84^ code. On the other hand, we have also employed the nonlocal vdW-DF and vdW-DF2 functionals,^85,86^ as implemented in the QE package,^77^ to further explore the influence of dispersion forces on the calculated properties. The exchange–correlation energy depends on the exchange energy, the correlation energy, and a term for the nonlocal electron correlation which is obtained using a double space integration, representing an improvement compared to local or semilocal functionals, as the vdW coefficients are themselves functionals of the electron density. The vdW-DF functional constitutes a first-principles DFT treatment of medium- and long-range interactions by means of a nonlocal density-based dispersion correction, while the vdW-DF2 variant involves changes to the exchange and nonlocal correlations to improve the estimation of the binding and counteract the effect of overbinding observed with vdW-DF.^35,38,85−87^

Once we checked the convergence, the energy cutoff for the plane-wave expansion of the wave functions was set up at 80 Ry (1088 eV) and charge density at 360 Ry (4898 eV). A Monkhorst–Pack 2 × 2 × 2 *k*-point grid^88^ in the reciprocal space was used per unit cell. Full geometry optimizations were performed by relaxing all atomic positions, employing the BFGS quasi-Newton algorithm with convergence criterion for the components of energy and forces being smaller than 0.0014 eV and 0.026 eV/Å, respectively. Regarding the isolated CO_2_ and H_2_O molecules, DFT calculations have been also performed at the Γ-point in a simulation cell of volume 30 × 30 × 30 Å^3^, considering the Makov–Payne method of electrostatic interaction correction for these aperiodic systems,^89^ to evaluate their energies.

The cohesive energies per water molecule for the fully single-occupied CO_2_@sH and the empty sH hydrates were computed as
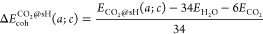
and
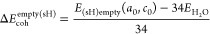
where *E*_CO_2_@sH_(*a*, *c*) and *E*_H_2_O_/*E*_CO_2__ are the total energies of the fully occupied CO_2_@sH hydrate unit cell with lattice constants *a* and *c* and of the isolated H_2_O/CO_2_ molecules, respectively, while *E*_(sH)empty_(*a*_0_, *c*_0_) is the total energy of the empty sH hydrate unit cell at the equilibrium lattice constants. In turn, the binding energy per CO_2_ molecule encaged in the empty sH is given by
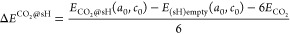


## Results and Discussion

### Structure H Hydrate Crystal Properties: Lattice Structure

Geometric optimizations of all atomic positions of the fully occupied CO_2_@sH and the empty sH unit cells were carried out to study the guest–lattice effects. DFT/DFT-D calculations without and with the dispersion correction were performed for fixed values of *r*, defined as the crystal atomic radius between the *c* and *a* lattice parameters (*r* = *c*/*a*) for the hexagonal sH structure.^63^ Total energies were computed as a function of the lattice constant *a* for the CO_2_@sH and sH hydrate clathrates, with the *r* and *a* ranges being 0.77–0.89 and 11.2–12.8 Å, respectively. The choice of the semilocal PW86PBE functional with the XDM or D4 correction is based on results reported from previous benchmark calculations on both clathrate-like clusters and sI/sII periodic systems,^44−46,48^ while the vdW-DF and vdW-DF2 functionals are also considered for exploring nonlocal dispersion effects.

**Figure 2:**
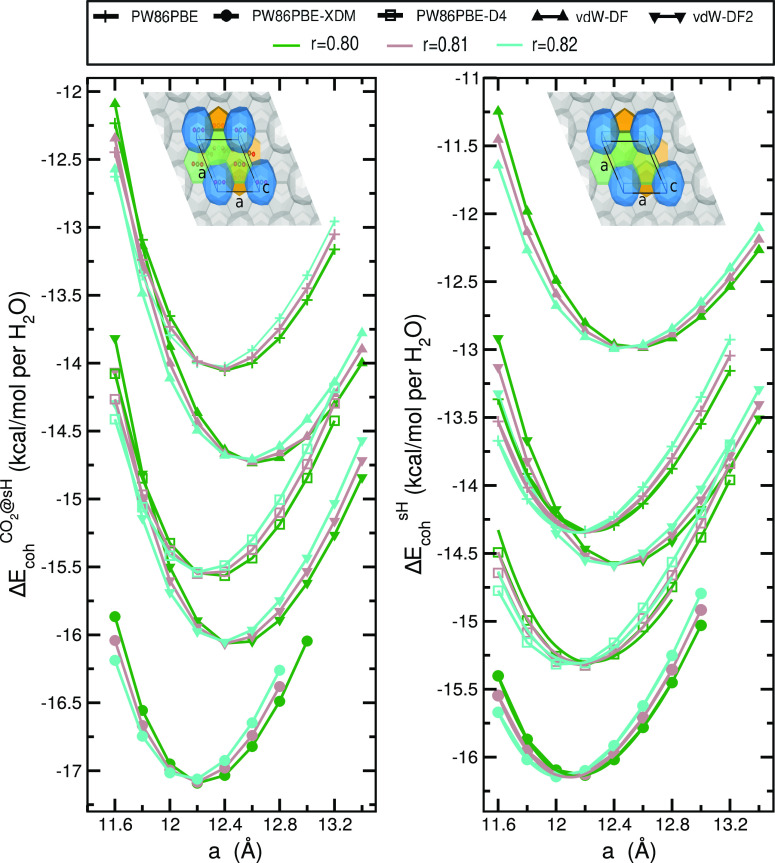
Cohesive energies of the fully occupied CO_2_@sH (right panel) and empty sH clathrate hydrate (left panel) as a function of lattice constant *a* and ratio *r*. Symbols indicate the computed values from the DFT/DFT-D periodic calculations using the QE code, while solid lines display their corresponding MEOS fits.

The equilibrium lattice parameters (*a*_0_ and *c*_0_) for the full CO_2_@sH and empty sH clathrate hydrates were calculated by fitting the values of the total CO_2_@sH(a) and empty sH(a) energies obtained from all-atom geometry relaxations as a function of *a* and *c* values to the Murnaghan equation of state (MEOS). The computed energy values are listed in Table S1, while in Figure S1 we displayed the total energy as a function of the volume of both CO_2_@sH and sH unit cells from PW86PBE, PW86PBE-XDM, and PW86PBE-D4, as well as vdW-DF and vdW-DF2 calculations. The cohesive energies are shown in Figure S2 for all *r* values, while in Figure 2 they are displayed for only three *r* values, as a function of the lattice parameter *a* for the fully occupied CO_2_@sH (right panel) and empty sH clathrate hydrate (left panel) considering the PW86PBE functional without and with XDM and D4 dispersion corrections and the vdW-DF and vdW-DF2 ones.

In the case of CO_2_@sH we found that the PW86PBE-XDM functional predicts more energetically favorable cohesive energies, reaching values near −17 (kcal/mol)/H_2_O, while the same functional without dispersion predicts less favorable values, near −14 (kcal/mol)/H_2_O, and vdW-DF and vdW-DF2 predict values around −14.7 and −16 (kcal/mol)/H_2_O, respectively.

The CO_2_@sH hydrate clathrate tends to achieve lower energies when noncovalent interactions are considered in the DFT calculations by implementing semiempirical dispersion corrections, considering the same functional with the XDM and D4 correction methods, or the nonlocal vdW-DF and vdW-DF2 functionals. The ratio *r* equal to 0.80 (green line in Figure 2, left panel) is the one that predicts the equilibrium structures for the three PW86PBE approaches studied, with *E*_coh_ = −17.092 (kcal/mol)/H_2_O, *a*_0_ = 12.239 Å, and *c*_0_ = 9.791 Å being the optimal parameters obtained for the CO_2_@sH unit cell with the PW86PBE-XDM functional, while *E*_coh_ = −16.075 (kcal/mol)/H_2_O with *a*_0_ = 12.487 Å and *c*_0_ = 9.990 Å are obtained from the vdW-DF2 calculations.

As there are no experimental reference data of lattice parameter values in the literature for the CO_2_@sH/sH clathrates, we choose to compare values available on similar systems. Thus, for example, powder X-ray diffraction (PXRD) data have confirmed the formation of binary sH hydrate containing molecular hydrogen in the small cages and large guest molecules in the large cavities, with the sH lattice parameters being *a*_0_ = 12.203 Å and *c*_0_ = 9.894 Å at 90 K,^49^ consistent with other sH hydrates for temperatures of 89–200 K^74,90^ or those (*a*_0_ = 11.980 Å and *c*_0_ = 9.992 Å) reported for CH_4_@sH at high pressures of 0.6–0.9 GPa.^66^ Another PXRD study on binary sH clathrate hydrates of neohexane (NH) with Ar, Kr, and CH_4_ at temperatures of 93–183 K has reported lattice parameters by means of thermal expansion extrapolation of *a* = 12.115 Å and *c* = 9.953 Å at *T* = 0 K^58^ in the case of Kr, resulting very closely to the PW86PBE-XDM MEOS fit values, within 1 and 1.6%, respectively. Further, more recent PXRD studies^7,91^ on sH clathrates containing nitrogen (N_2_) and NH molecules have estimated unit cell dimensions to be *a*_0_ = 12.2342 Å and *c* = 9.9906 Å at 153 K, while the lattice parameters of the sH hydrates formed in CO_2_ (20%) + N_2_ (80%) + NH gas mixtures have been reported to be *a*_0_ = 12.25 and *c* = 10.14 Å. Our results are in good accord with these estimates, presenting errors of less than 0.3 and 1% for *a* and *c*, respectively, compared to the later PXRD data available.

For the empty sH we found similar results (see right panel in Figure 2), with PW86PBE-XDM predicting the lowest cohesive energy values near −16 (kcal/mol)/H_2_O, PW86PBE-D4 values are found near −15.5 (kcal/mol)/H_2_O while the same functional without dispersion predicts less favorable values by about 2 (kcal/mol)/H_2_O, and the vdW-DF and vdW-DF2 values are around −13 and −14.5 (kcal/mol)/H_2_O, respectively. The ratio *r* value of 0.82 (see cyan line in Figure 2) is the one that predicts the equilibrium structure considering the three DFT/DFT-D approaches, with the optimal lattice sH parameters being *a*_0_ = 12.038 Å and *c*_0_ = 9.871 Å, and *E*_coh_= −16.143 (kcal/mol)/H_2_O, from the PW86PBE-XDM calculations. The optimal *a*_0_ and *c*_0_ values of 12.447/12.352 and 10.207/10.128 Å, obtained from the vdW-DF/vdW-DF2 calculations, are larger than those from PW86PBE. Once more, due to the lack of experimental references of the equilibrium lattice constants, *a* and *c*, for the sH empty hydrate, we have compared our results with those of refs (63, 70, 73, and 74) as given from single crystal X-ray diffraction experiments and MD simulations on similar sH hydrate systems, as well as DFT calculations with B3LYP, rev-PBE, and vdW-DRSLL functionals for the sH hydrate. The reported values from the later DFT study^63^ for the sH lattice constants were *a* = 12.100 Å and *c* = 9.960 Å, which are close to the present PW86PBE-XDM results within 0.1 Å and around 0.3 Å compared to the vdW-DF/vdW-DF2 data in both cases.

Another representation of the variation of the cohesive energy as a function of the lattice parameter *a* and the ratio *r* is shown in Figure S3, where contour plots for CO_2_@sH and sH clathrate hydrates considering PW86PBE functional without and with XDM and D4 dispersion corrections are displayed. The dark red color corresponds to cohesive energy minima, while dark blue areas correspond to higher energy values. The equipotential lines are plotted according to the maximum and minimum of each case with intervals of 0.2 (kcal/mol)/H_2_O. It is possible to see the comparison between the fully filled CO_2_@sH clathrate and the empty sH hydrate unit cells for the different DFT-D functionals. In all cases, the minimum corresponds to *r* values of 0.8–0.81 and 0.81–0.82 for the filled clathrate and empty sH hydrates, respectively.

The structural parameters obtained from the corresponding MEOS fits for each functional are listed in Table 1. As shown, the PW86PBE-XDM functional predicts the most stable cohesive energies for both fully CO_2_-filled and empty sH crystal hydrate, reaching energies of −17 and −16 kcal/mol, respectively, contrary to the case of the functional without dispersion that does not take into account the vdW forces, specifically in the guest–host interactions, and it predicts energies about 17% higher than those obtained when considering the XDM dispersion scheme. Further, we found that the absence of CO_2_ molecules trapped inside the crystal lattice implies a structural change represented by a reduction in the *a*_0_ lattice constants considering the three variants of the PW86PBE functional, as well as the vdW-DF and vdW-DF2 ones. Concerning the *c*_0_ lattice parameter values, we found that PW86PBE-XDM and vdW-DF2 predict a reduction of this constant when the hydrate is filled, while the opposite, an increase in the *c* lattice parameter, is observed in all other cases.

**Table 1:** Parameters Obtained from MEOS Fit by Considering PW86PBE Functional without and with XDM or D4 Correction Dispersion and Nonlocal vdW-DF and vdW-DF2 Functionals for CO_2_@sH and sH Clathrate Hydrates

hydrate	parameter	PW86PBE	PW86PBE-XDM	PW86PBE-D4	vdW-DF	vdW-DF2
CO_2_@sH	*a*_0_ (Å)	12.424	12.239	12.328	12.604	12.487
	*c*_0_ (Å)	9.939	9.791	9.863	10.209	9.990
	*r*_0_	0.8	0.8	0.8	0.81	0.8
	*V*_0_ (Å^3^)	1328.549	1270.130	1298.142	1404.361	1348.886
	*B*_0_ (GPa)	11.91	14.38	12.57	9.84	12.03
	*B*_0_′	5.26	5.47	5.85	4.92	5.70
sH	*a*_0_ (Å)	12.241	12.038	12.129	12.447	12.352
	*c*_0_ (Å)	9.915	9.871	9.825	10.207	10.128
	*r*_0_	0.81	0.82	0.81	0.82	0.82
	*V*_0_ (Å^3^)	1270.731	1238.903	1251.779	1369.463	1338.204
	*B*_0_ (GPa)	11.17	12.56	11.82	7.88	9.83
	*B*_0_′	5.73	5.99	6.20	5.51	5.53

### Pressure Effects and Compressional Anisotropy

**Figure 3:**
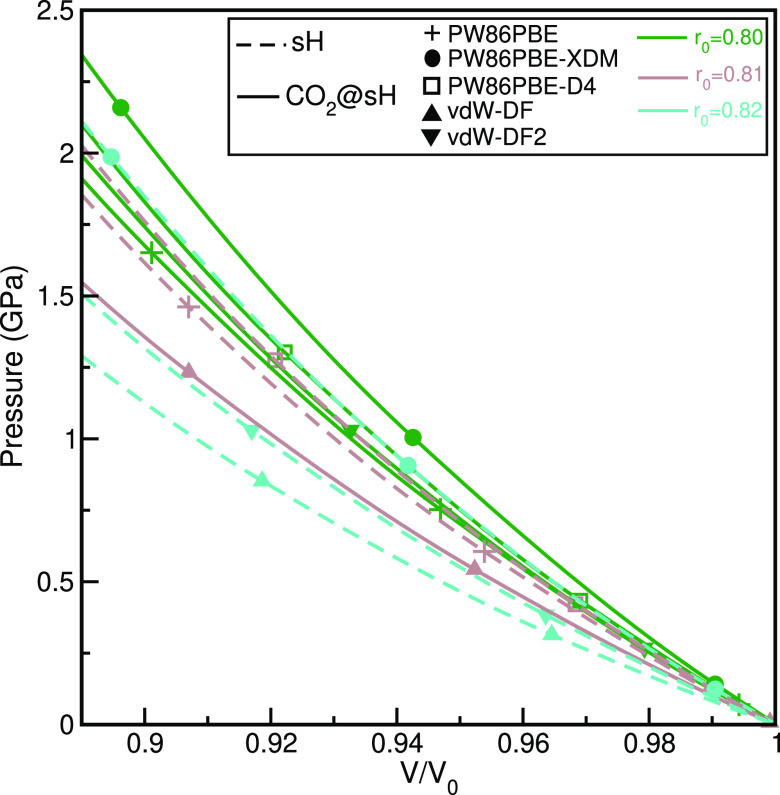
Pressure effects on unit cell volumes of CO_2_@sH clathrate and empty sH hydrate. Symbols correspond to the computed values from the indicated DFT/DFT-D periodic calculations using the QE code, while solid lines display their corresponding MEOS fits.

The effects of pressure on the structures of CO_2_@sH and sH clathrate hydrates are also investigated, as described quantitatively by the MEOS. The pressure–volume diagrams, shown in Figure 3, were obtained from the MEOS equation
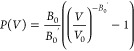
for both the CO_2_-filled and empty sH hydrates at *T* = 0 K. Results are given up to a high pressure of 2.5 GPa, which is beyond the CO_2_ hydrate decomposition.^2^

As can be seen in Figure 3, the volume is compressed somehow more rapidly for the empty sH hydrate than for the CO_2_@sH clathrate when pressure is increased, according to the results from the PW86PBE-XDM, vdW-DF2, and vdW-DF calculations. A similar behavior is also predicted from the PW86PBE-D4 and PW86PBE data, although the differences between the curves corresponding to the empty and single CO_2_-filled sH structures are smaller, compared to those obtained from the dispersion-corrected PW86PBE-XDM, vdW-DF2, and vdW-DF functionals. In Table 1 we list the equilibrium unit cell volume (*V*_0_), the isothermal bulk modulus *B*_0_, and the bulk modulus pressure derivative *B*_0_^′^ at zero pressure, as estimated from the fittings to the MEOS equation, for CO_2_@sH clathrate and empty sH hydrate from all PW86PBE/-XDM/-D4 calculations. The MEOS equation assumes linear dependence of the bulk modulus with pressure, *B* = *B*_0_ + *B*_0_^′^*P*, with *B*_0_ constant, and thus is valid for low compressions with 0 < *P* < *B*_0_^′^/2.

From PW86PBE/-XDM/-D4 (without/with dispersion corrections) calculations we obtained *B*_0_ values of 11.91/14.38/12.57 GPa and *B*_0_^′^ = 5.26/5.47/5.85 for the bulk CO_2_@sH clathrate and *B*_0_ = 11.17/12.56/11.82 GPa and *B*_0_^′^ = 5.73/5.99/6.20 for the sH empty hydrate, while from vdW-DF/vdW-DF2 *B*_0_ values of 9.84/12.03 and 7.88/9.83 GPa and *B*_0_^′^ values of 4.92/5.70 and 5.51/5.53 were calculated for the bulk CO_2_ filled and empty sH hydrates. All DFT/DFT-D functionals predict larger *B*_0_ values for CO_2_@sH than the empty sH hydrate, with PW86PBE-XDM yielding higher *B*_0_ values (lower compressibility) than those obtained from the remaining PW86PBE, PW86PBE-D4, and nonlocal vdW-DF/vdW-DF2 functionals. The higher *B*_0_ values reflect the effect of the vdW dispersion interactions and are consistent with the higher binding found from the PW86PBE-XDM calculations compared to those with the PW86PBE functional, in accord with previous studies in similar clathrate systems.^63,92^ In turn, we found that the *B*_0_ values for the CO_2_@sH and sH crystals are higher than those computed for the bulk CO_2_@sI, sI and sII and smaller compared to the bulk CO_2_@sII, according to the PW86PBE-XDM results from refs (45 and 46). Such comparisons indicate that the CO_2_@sH systems show less and more resistance to compression than CO_2_@sI and CO_2_@sII, respectively.

**Figure 4:**
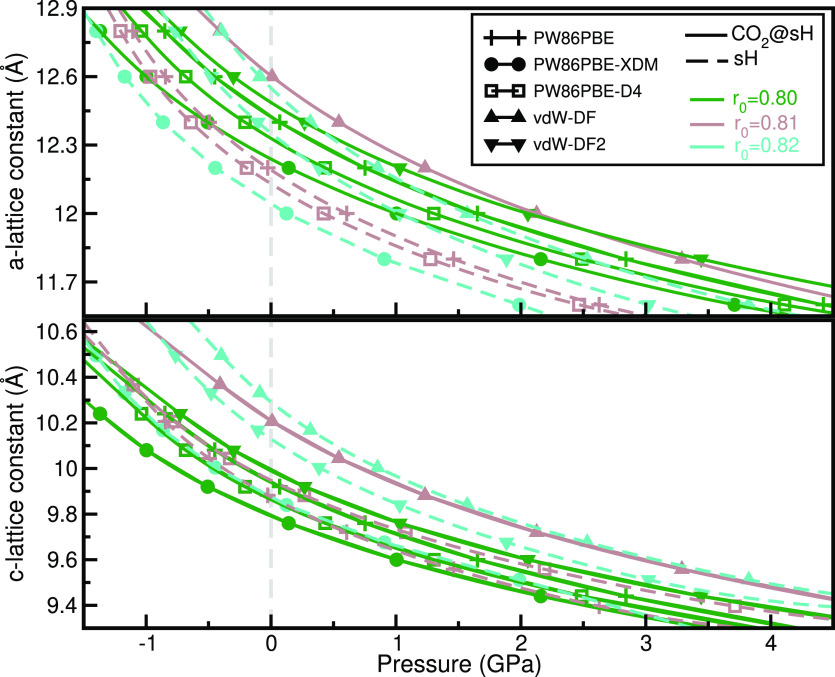
Pressure effect on *a* and *c* lattice parameters for CO_2_@sH and sH hydrates. Symbols correspond to the computed values from the indicated DFT/DFT-D functional. Solid lines correspond to fully occupied CO_2_@sH hydrate, and dashed lines correspond to the empty sH hydrate.

As the sH hydrates are identified by structural anisotropy due to their hexagonal lattice structure, we have also analyzed and compared it to the relatively isotropic cubic sI and sII hydrate structures. Thus in Figure 4 we display the compressional response of the *a* and *c* lattice constants of CO_2_@sH and sH, where their changes as a function of pressure at 0 K are shown from the PW86PBE/-XDM/-D4 and vdW-DF/vdW-DF2 calculations. One can see that the *a* lattice constant presents higher changes than the *c* lattice constant, indicating that both CO_2_@sH clathrates and sH hydrates are more compressible in the *a* direction than in the *c* one. The *a* lattice constant increases with the CO_2_ filling of the sH hydrate, while the *c* lattice constant decreases, except the PW86PBE case. The PW86PBE and PW86PBE-D4 as well as the vdW-DF and vdW-DF2 results show similar changes in lattice parameters with pressure, while the PW86PBE-XDM data predict more compact and less compressible structures (except in the *c* direction of the empty sH structure systems). The same behavior in the *a* lattice constant with pressure has been also observed in the cases of the CO_2_@sI and CO_2_@sII clathrates and empty sI and sII hydrates. Such an anisotropic lattice expansion of the sH hydrates has been previously reported for several binary sH clathrates^58,63^ and has been found to affect their thermal expansion,^90^ too.

### Structure H Energetics: Gradual CO_2_@sH Cage Occupation

**Figure 5:**
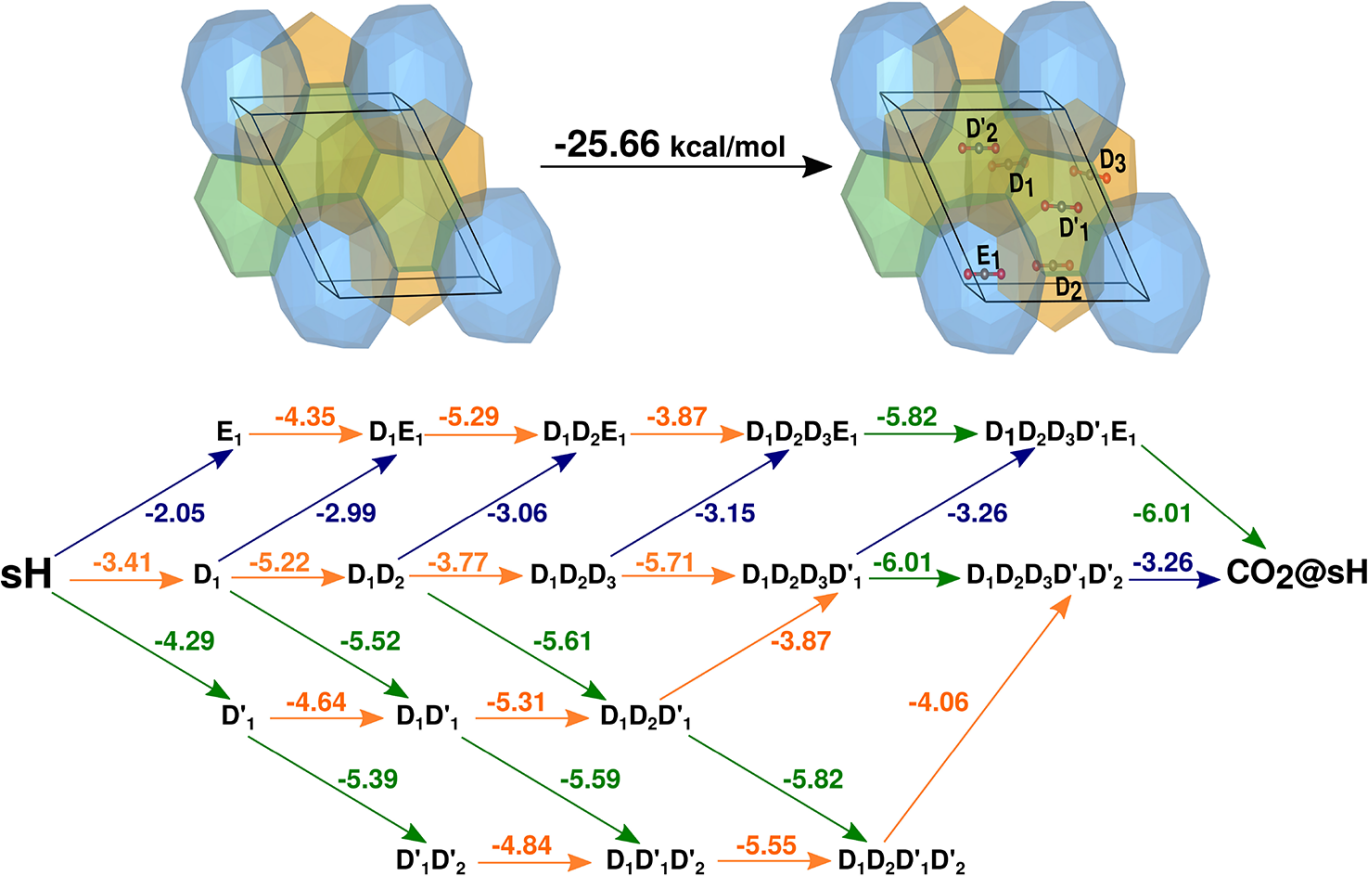
Schematic representation of the gradual single CO_2_ cage occupation of the sH crystal. Yellow, green, and blue correspond to the D, D′, and E cages, respectively, and energies (in kcal/mol) at each indicated step from PW86PBE-XDM calculations.

In the case of single cage occupancy, we present the results on the energetics focused first on the study on progressive CO_2_ occupancy of the sH crystal unit cell. We start from the empty sH unit cell, and in each step the system is optimized by including one more CO_2_ molecule in each of the remaining sH cages. In this way, the binding energies with respect to the guest-free or partially filled system were calculated, and in Figure 5 all such single filling processes are shown, starting from one up to six CO_2_ guests.

In Figure 5, we labeled the six CO_2_ positions in the CO_2_@sH unit cell. Thus, positions 1–3 correspond to the small D cages (D_1_, D_2_, D_3_), while positions 4 and 5 correspond to D′ (D′_1_, D′_2_) cages and position 6 corresponds to the large E (E_1_) sH cage. The results of the energetics shown in Figure 5 correspond to those obtained from the PW86PBE-XDM calculations. The numbers on each arrow in Figure 5 indicate the energy gained in the corresponding step of the process with respect to the empty or partially occupied sH clathrate and the free CO_2_ molecules. The obtained results show that CO_2_ binding is more favorable for the D′ cage, with an energy gain between 4.29 and 6.01 kcal/mol at each step, indicating a preference of the CO_2_ for occupying the irregular D′ cage of the sH hydrate as well as the D cages, estimating average energies of −4.13, −4.84, and −2.05 kcal/mol for the CO_2_ enclathration in the D, D′, and E sH crystal cages, respectively. According to our PW86PBE-XDM computations the energy difference between the occupations of the D and irregular D′ cages is around 0.7 kcal/mol, while the energy difference between the occupations of the D′ and E cages is found to be 2.8 kcal/mol. It is clear that there is a significant preference of the CO_2_ for the D′ and D cages compared to the E cage. We found that the full single CO_2_ occupation of the sH structure is energetically favorable, with a total energy gain of 25.655 kcal/mol from the PW86PBE-XDM calculations. Such a preference of the CO_2_ molecule for occupying a specific type/size of cage has been previously reported for the T cage of the CO_2_@sI clathrate crystal,^16,25,44,45,48^ which has been related to the CO_2_ orientation in the cage and the stability of this clathrate.

In Table 2 we also present results on the energetics from all present DFT/DFT-D calculations on the saturation energy of the fully single CO_2_ filled CO_2_@sH clathrate hydrate. One can see that full occupation is favorable only when the XDM or D4 dispersion corrections are considered in the semilocal PW86PBE functional. The more energetically stable value is obtained with the PW86PBE-XDM functional, with a mean energy gain of 4.28 (kcal/mol)/CO_2_ compared to the 0.56 (kcal/mol)/CO_2_ from the PW86PBE-D4 computations. In turn, both nonlocal vdW-DF and vdW-DF2 functionals predict even more favorable CO_2_ occupation of the sH structure with mean energy gains of 8.74 and 7.49 (kcal/mol)/CO_2_, respectively. The sH structure is the smallest in size compared to sI and sII hydrates, with its unit cell containing 34 water molecules forming 6 cages, close to the size of the sI with 46 water molecules and 8 cages, while the sII unit cell is larger with 136 water molecules and 24 cages. Therefore, regarding their energetic stability at zero pressure and temperature, we found that it decreases in the sequence CO_2_@sI, CO_2_@sII, and CO_2_@sH clathrates, with mean occupation energies of −6.375, −4.471, and −4.28 (kcal/mol)/CO_2_, respectively, from the PW86PBE-XDM calculations,^45,46^ with the single fully CO_2_-filled sH structure being the less stable one.

**Table 2:** Saturation Energy for the Fully Single CO_2_ Filled sH Hydrate, Binding Energy per CO_2_ to the Empty sH, and Cohesive Energy of the Empty sH hydrate Computed from the Indicated DFT and DFT-D Calculations

energy	PW86PBE	PW86PBE-XDM	PW86PBE-D4	vdW-DF	vdW-DF2
*ΔE*_sat_ (kcal/mol)	14.283	–25.655	–3.267	–52.426	–44.946
*ΔE*^CO_2_@sH^ ((kcal/mol)/CO_2_)	–9.287	–18.017	–14.597	–17.612	–16.238
*ΔE*_coh_^empty(sH)^ ((kcal/mol)/H_2_O)	–12.417	–13.915	–13.003	–11.618	–13.210

The binding energy of the CO_2_ to empty sH and the cohesive energy of empty sH hydrate at their corresponding equilibrium lattice parameters *a*_0_ and *c*_0_ obtained from the present calculations are also listed in Table 2. We found that PW86PBE-XDM, PW86PBE-D4, and vdW-DF2 calculations predict close cohesive energy values of −13.915, 13.003, and −13.210 (kcal/mol)/H_2_O, respectively, while PW86PBE-XDM predicts a stronger binding of CO_2_ compared to PW86PBE-D4 as well as to the PW86PBE functional without dispersion, with values of −18.017, −14.597, and −9.287 (kcal/mol)/CO_2_, respectively, indicating clearly the effect of the dispersion corrections. Similar values of −17.612 and −16.238 (kcal/mol)/H_2_O are also obtained by employing the nonlocal vdW-DF and vdW-DF2 functionals.

### CO_2_ Orientation in the sH Crystal: Comparative Analysis

**Figure 6:**
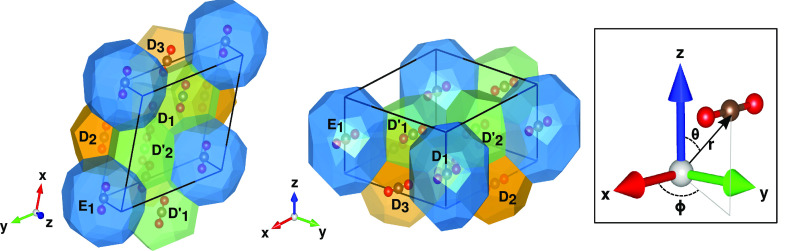
Views of the orientation of the CO_2_ molecules inside the fully occupied sH unit cell obtained from periodic PW86PBE-XDM calculations.

In Figure 6 we display different *xyz* views of the fully occupied CO_2_@sH crystal’s unit cell as obtained from the PW86PBE-XDM geometry relaxation calculations. As CO_2_ is a linear molecule, we discuss its orientation in the cages in terms of (*r*, θ, ϕ) coordinates, as shown in the inset plot of Figure 6, where *r* is the distance of the C atom from the center of each cage, while θ and ϕ are the polar and azimuthal angles, respectively. The corresponding coordinates for each encapsulated CO_2_ in the D, D′, and E sH cages are listed in Table S2.

As it is seen, θ angle values are close to 90° (between 88.4 and 89.8°) in all cases, while ϕ ranges between 0.37 and 9.4° in the CO_2_@sH unit cell crystal. The CO_2_ molecules are found to be located almost at the center of the D′ and E cages (shifted by just 0.05 and 0.004 Å), while off-center shifts by 0.15–0.25 Å are obtained for the D cages. By examining the CO_2_ orientations trapped in the individual D, D′, and E cages reported in previous DF-MP2 and DFT/B3LYP-D3M(BJ) finite-size cluster calculations,^25^ we found that CO_2_ in the large E cage maintains its orientation in the periodic system, while changes are observed in its orientation inside the smaller D and D′ cages compared to the individual cluster systems. In particular, the CO_2_ molecules show an alignment in the unit cell crystal cages, in contrast to the individual cage cases. With respect to the CO_2_ position in each sH cage, we found some differences between the values in the individual cages and those in the unit cell crystal. We obtained that the CO_2_ molecule is located slightly offset from the center of the D cage in the periodic system, while in the individual cage this shift is not observed. In the case of the D′ and E cages, the shifts are smaller in the periodic unit cell than those in the individual cages. As discussed, during the progressive single CO_2_ occupation an energetic preference in occupying the D′ and D cages of the sH crystal cell compared to the E cage is found; however, such a preference clearly depends on the type/size of the cage, as well as its position and surrounding connections in the crystal unit cell.

## Summary and Conclusions

We have carried out first-principles computations for the entire periodic crystal of the CO_2_@sH clathrate hydrate for evaluating guest–lattice effects and their influence on the crystal structure and energetics. In particular, we started by benchmarking the performance of semilocal and nonlocal DFT/DFT-D functionals, identifying those that performed best, and then employed them to investigate the structural stability and energetics of the single CO_2_ gradual occupation process of the sH crystal. Our results on binding and cohesion energies, lattice parameters, and pressure compressibility effects indicate the importance of including dispersion corrections in both empty sH hydrate and CO_2_@sH clathrate hydrate crystals. On the basis of our analysis the PW86PBE functional with XDM dispersion correction yields the best performance on the interaction between the CO_2_ guest and the sH lattice, in accord with previous studies in sI and sII crystals, while the results of the nonlocal vdW-DF and vdW-DF2 functionals reflect an expansion in the unit cell parameters with variant response to pressure in both CO_2_@sH and sH crystals.

The present computations reveal that the process of CO_2_ enclathration in the sH structure is energetically favorable, with a total energy gain of near 26 kcal/mol from the PW86PBE-XDM calculations, for the fully single CO_2_ occupation of the sH unit cell, which is less than the energy gain in the cases of CO_2_@sI and CO_2_@sII clathrates. We also observed structural changes when CO_2_ molecules get trapped in the sH crystal lattice, represented by an increase in the *a* lattice constant and a reduction in the *c* lattice constant compared to the empty sH crystal. Pressure effects were also evaluated, and compressional anisotropy was found to be noticeable by comparing the response of the *a* and *c* lattice parameters as a function of pressure. The occupancy preferences of CO_2_ in the sH crystal were also investigated, and it was found that the D′ cages are more favored than the D or E cages. Our results confirm that the CO_2_ molecules show an aligned orientation in the sH crystal cages, with the energetic preference in filling the D′ and D cages compared to the E depending on the size of the cage and its surrounding connections in the crystal lattice.

The computations also show the complex interplay of the details of the molecular structure and energetics in determining lattice parameters and stability of single molecule clathrate hydrates. In cases where cages have multiple CO_2_ or binary guest occupancies, other complications will enter for predicting lattice constants and phase stability. Thus, due to the high relevance in the field of CO_2_ gas sequestration and storage applications, the impact of multiple cage occupancies on the stability of CO_2_@sI/sII/sH clathrates and the presence of a help gas for enhancing capacity storage will be the next issues to explore, looking into evolutions that lead to stabilizing effects of sI/sII/sH structures when the CO_2_ content increases. Such a structural stability factor analysis for multicomponent systems requires a detailed and reliable description of the bonding and nonbonding contributions to the potential guest–host, host–host, and guest–guest molecular interactions, considering lattice effects in the complete unit cell, followed by a complete thermodynamic investigation under pressure and temperature.

Overall, our present results contribute to enhance understanding of the underlying interactions that serve to check and test earlier assumptions, leading to computationally improved estimates and proper characterization of the material properties of sH hydrate crystals. We envisage that the outcome of the present first-principles study will trigger further investigations on identifying formation processes and mechanisms, highly related to gas hydrate based CO_2_ capture technologies.

## Supporting Information Available

MEOS fit parameters and CO_2_ orientations in the CO_2_@sH crystal unit cell from DFT-D calculations; energies versus volume and lattice constant dependence (PDF)
