# Supplementary material for: Modeling of Structure H Carbon Dioxide Clathrate Hydrates: Guest–Lattice Energies, Crystal Structure, and Pressure Dependencies
Source: J Phys Chem C Nanomater Interfaces. 2022 Aug 26;126(35):14832–42. doi: 10.1021/acs.jpcc.2c04140 (PMC9465682; doi:10.1021/acs.jpcc.2c04140)
Supplement: Supplementary file 1 — jp2c04140_si_001.pdf [file jp2c04140_si_001.pdf]

Modeling of Structure H Carbon Dioxide Clathrate Hydrates: Guest-Lattice Energies, Crystal Structure and Pressure Dependencies

Adriana Cabrera-Ramírez[†,‡] and Rita Prosmiti[*,†]

†*Institute of Fundamental Physics (IFF-CSIC), CSIC, Serrano 123, 28006, Madrid, Spain*

‡*Doctoral Programme in Theoretical Chemistry and Computational Modelling, Doctoral School, Universidad Autónoma de Madrid, Madrid, Spain*

E-mail: rita@iff.csic.es

Supporting Information Available

Table S1: Parameters obtained from Murnaghan's equation of state (MEOS) fit by considering the PW86PBE functional without and with XDM/D4 semiempirical dispersion corrections, and the nonlocal vdW-DF and vdW-DF2 functionals for the CO_2@sH and sH clathrate hydrates.

r	**CO2@sH**						**sH**					
	Parameter	PW86PBE	PW86PBE-XDM	PW86PBE-D4	vdW-DF	vdW-DF2	Parameter	PW86PBE	PW86PBE-XDM	PW86PBE-D4	vdW-DF	vdW-DF2
0.77	E_0 (eV)	-28864.09274	-28869.09379	-28866.75426	-	-	E_0 (eV)	-20506.39903	-20509.36731	-20508.06211	-	-
	a_0 (Å)	12.56525	12.380264	12.468605	-	-	a_0 (Å)	12.399248	12.2964	12.340565	-	-
	V_0 (Å^3)	1322.884671	1265.313941	1292.564243	-	-	V_0	1271.143855	1239.774089	1253.180988	-	-
	B_0 (GPa)	12.15524	14.59033	13.103844	-	-	B_0	11.252423	12.417386	11.874944	-	-
	B_0'	5.177284	5.379344	5.495612	-	-	B_0'	5.283876	5.343122	5.375777	-	-
	c_0 (Å)	9.6752425	9.53280328	9.60082585	-	-	c_0 (Å)	9.54742096	9.468228	9.50223505	-	-
0.8	E_0 (eV)	-28864.11513	-28869.10419	-28866.74022	-29120.74442	-29356.34966	E_0 (eV)	-20506.4661	-20509.44464	-20508.12609	-20684.31479	-20850.15003
	a_0 (Å)	12.423862	12.239023	12.328344	12.64464	12.486934	a_0 (Å)	12.242453	12.142219	12.181461	12.553932	12.459272
	V_0	1328.549366	1270.129963	1298.141856	1400.642441	1348.886177	V_0	1271.198146	1240.229508	1252.293105	1370.715252	1339.941677
	B_0	11.911024	14.381448	12.574798	9.747148	12.03177	B_0	11.143961	12.456026	11.729128	7.935001	9.829089
	B_0'	5.258117	5.47164	5.85095	5.152686	5.696195	B_0'	5.626518	5.655718	6.055842	5.306825	5.273727
	c_0 (Å)	9.9390896	9.7912184	9.8626752	10.115712	9.9895472	c_0 (Å)	9.7939624	9.7137752	9.7451688	10.0431456	9.9674176
0.81	E_0 (eV)	-28864.10117	-28869.08817	-28866.72097	-29120.74761	-29356.33537	E_0 (eV)	-20506.4758	-20509.45599	-20508.13659	-20684.31936	-20850.15722
	a_0 (Å)	12.376014	12.186682	12.281722	12.603522	12.438214	a_0 (Å)	12.240955	12.090003	12.129463	12.500174	12.405093
	V_0	1329.674276	1269.577856	1299.513079	1404.360864	1349.823448	V_0	1270.73139	1239.601664	1251.778976	1370.096277	1339.068947
	B_0	11.736667	14.318643	12.54021	9.837021	11.99123	B_0	11.171379	12.508608	11.816098	7.894956	9.828682
	B_0'	5.450587	5.897893	5.897428	4.923677	5.67444	B_0'	5.730149	5.833419	6.20395	5.424232	5.409649
	c_0 (Å)	10.02457134	9.87121242	9.94819482	10.20885282	10.07495334	c_0 (Å)	9.91517355	9.79290243	9.82486503	10.12514094	10.04812533
0.82	E_0 (eV)	-28864.07926	-28869.06674	-28866.6915	-29120.71244	-29356.31475	E_0 (eV)	-20506.47485	-20509.45818	-20508.13405	-20684.32124	-20850.15799
	a_0 (Å)	12.329013	12.142354	12.233489	12.545861	12.389539	a_0 (Å)	12.138391	12.038392	12.080235	12.447235	12.3518
	V_0	1330.81179	1271.277628	1330.117882	1402.274841	1350.507943	V_0	1270.033386	1238.902751	1251.866206	1369.463255	1338.204402
	B_0	11.670992	14.387119	12.533751	9.752544	11.999413	B_0	11.168111	12.556265	11.730647	7.882657	9.827005
	B_0'	5.62094	5.829689	6.267929	5.254335	5.655414	B_0'	5.86998	5.990812	6.24322	5.506226	5.530764
	c_0 (Å)	10.10979066	9.95673028	10.03146098	10.28760602	10.15942198	c_0 (Å)	9.95348062	9.87148144	9.9057927	10.2067327	10.128476
0.83	E_0 (eV)	-28864.05052	-28869.02961	-28866.65803	-	-	E_0 (eV)	-20506.47027	-20509.45565	-20508.13204	-	-
	a_0 (Å)	12.282977	12.09561	12.189411	-	-	a_0 (Å)	12.088036	11.988538	12.031468	-	-
	V_0	1332.008313	1271.977181	1301.7995320	-	-	V_0	1269.589297	1238.495994	1251.848749	-	-
	B_0	11.625026	14.216617	12.506114	-	-	B_0	11.211921	12.618697	11.893895	-	-
	B_0'	5.504992	5.793518	5.993407	-	-	B_0'	5.909329	6.035533	6.042432	-	-
	c_0 (Å)	10.19487091	10.0393563	10.11721113	-	-	c_0 (Å)	10.03306988	9.95048654	9.98611844	-	-
0.84	E_0 (eV)	-28864.01145	-28868.9943	-28866.61217	-	-	E_0 (eV)	-20506.45652	-20509.44347	-20508.12155	-	-
	a_0 (Å)	12.235049	12.048371	12.144166	-	-	a_0 (Å)	12.037247	11.937899	11.994546	-	-
	V_0	1332.337548	1272.278491	1302.867525	-	-	V_0	1268.757792	1237.601732	1255.303138	-	-
	B_0	11.628339	14.453889	12.577284	-	-	B_0	11.211362	12.661321	11.547839	-	-
	B_0'	5.721189	6.050716	6.177515	-	-	B_0'	6.100196	6.227649	5.045131	-	-
	c_0 (Å)	10.27744116	10.12063164	10.20109944	-	-	c_0 (Å)	10.11128748	10.02783516	10.07541864	-	-
0.85	E_0 (eV)	-28863.96732	-28868.94037	-28866.562	-	-	E_0 (eV)	-20506.43469	-20509.42518	-20508.09688	-	-
	a_0 (Å)	12.192033	12.003286	12.101409	-	-	a_0 (Å)	11.986517	11.887262	11.931097	-	-
	V_0	1334.028765	1273.026122	1304.501548	-	-	V_0	1267.698007	1236.466534	1250.195434	-	-
	B_0	11.741001	14.405166	12.62239	-	-	B_0	11.224292	12.784707	11.956682	-	-
	B_0'	5.424619	6.130072	6.054415	-	-	B_0'	6.251592	6.46304	6.346401	-	-
	c_0 (Å)	10.36322805	10.2027931	10.28619765	-	-	c_0 (Å)	10.18853945	10.1041727	10.14143245	-	-
0.86	E_0 (eV)	-28863.91754	-28868.88686	-28866.50682	-	-	E_0 (eV)	-20506.40871	-20509.39813	-20508.07094	-	-
	a_0 (Å)	12.144407	11.960627	12.056415	-	-	a_0 (Å)	11.938082	11.838763	11.882953	-	-
	V_0	1333.967451	1274.319136	1305.181465	-	-	V_0	1267.126619	1235.763379	1249.653268	-	-
	B_0	11.89927	14.541794	12.777946	-	-	B_0	11.246467	12.760918	12.000083	-	-
	B_0'	5.609044	5.654925	6.243417	-	-	B_0'	6.273202	6.431677	6.389156	-	-
	c_0 (Å)	10.32274595	10.16653295	10.24795275	-	-	c_0 (Å)	10.26675052	10.18133618	10.21933958	-	-
0.89	E_0 (eV)	-28863.70201	-28868.68385	-28866.2971	-	-	E_0 (eV)	-20506.26128	-20509.26149	-20507.93346	-	-
	a_0 (Å)	11.987286	11.80601	11.891813	-	-	a_0 (Å)	11.795912	11.659009	11.732701	-	-
	V_0	1327.610049	1268.286286	1296.140651	-	-	V_0	1265.034794	1221.49809	1244.806698	-	-
	B_0	11.71641	15.217149	12.966219	-	-	B_0	8.548671	8.704154	9.94223	-	-
	B_0'	6.306894	7.02509	8.774965	-	-	B_0'	1.22865	1.341356	3.305359	-	-
	c_0 (Å)	10.66868454	10.5073489	10.58371357	-	-	c_0 (Å)	10.49836168	10.37651801	10.44210389	-	-

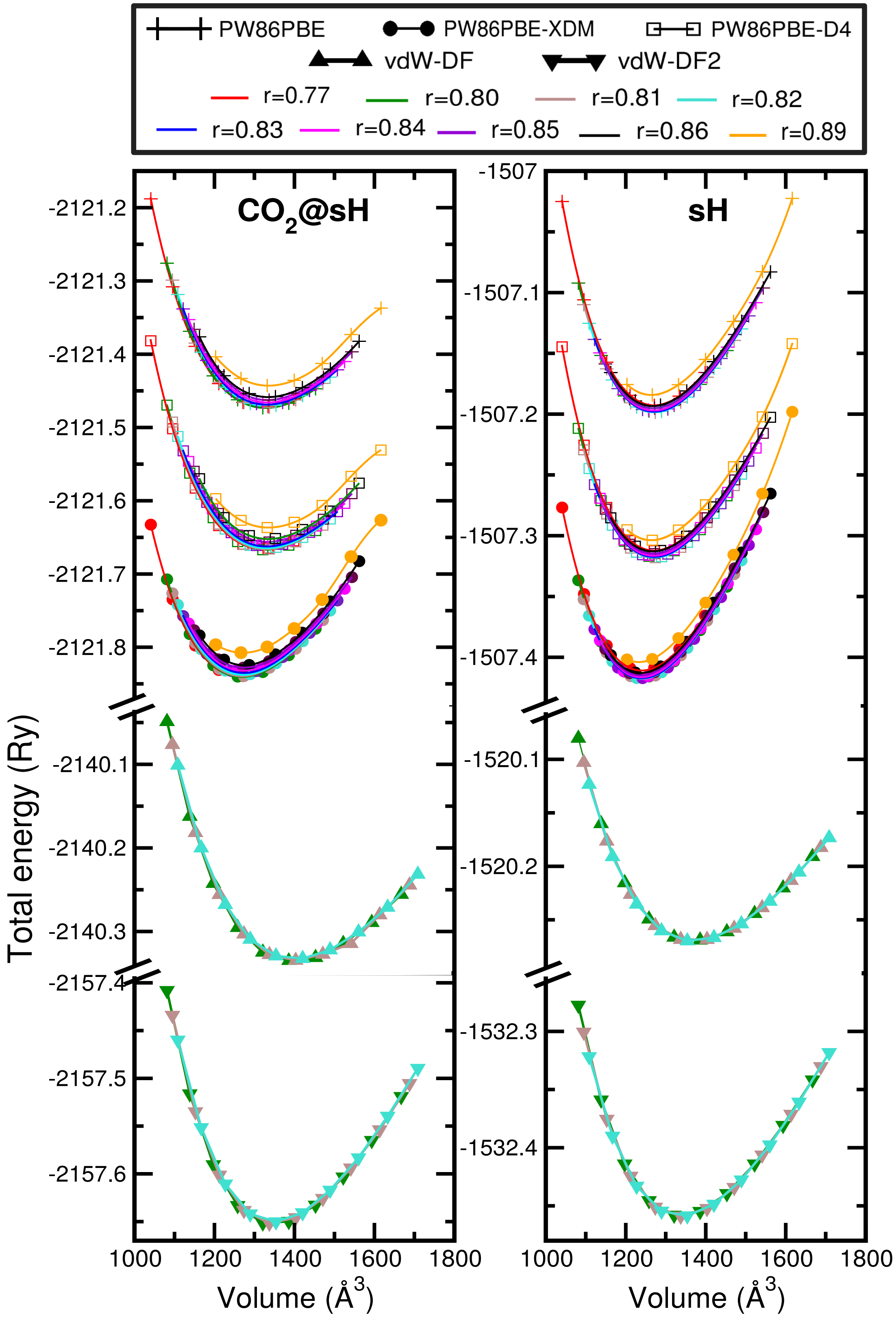

Figure S1: Energy vs volume plots for the CO_2@sH and sH clathrate hydrates obtained from the indicated DFT/DFT-D calculations (see symbols), together with the corresponding MEOS fits (see solid lines).

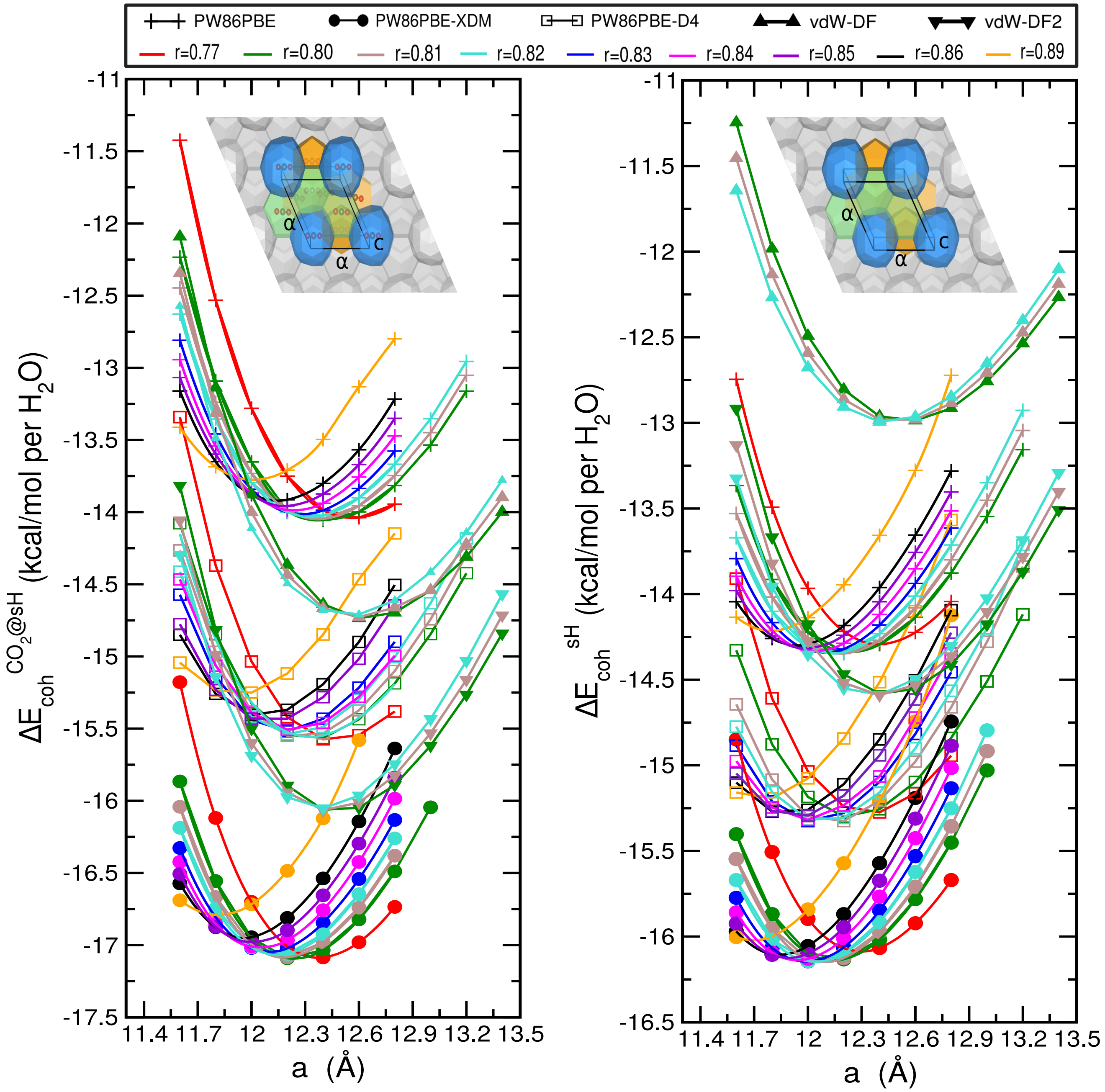

Figure S2: Cohesive energies of the fully occupied CO_2@sH (right panel) and empty sH clathrate hydrate (left panel) as a function of the lattice constant a and ratio r. Symbols indicate the computed values from the DFT/DFT-D periodic calculations using the QE code, while solid lines display their corresponding MEOS fits.

Figure S3: Contour plots of the cohesive energy (in kcal/mol/H_2O) in the (r,a)-plane for the full filled CO_2@sH and empty sH clathrates by considering the PW86PBE functional without and with the XDM or D4 dispersion corrections.

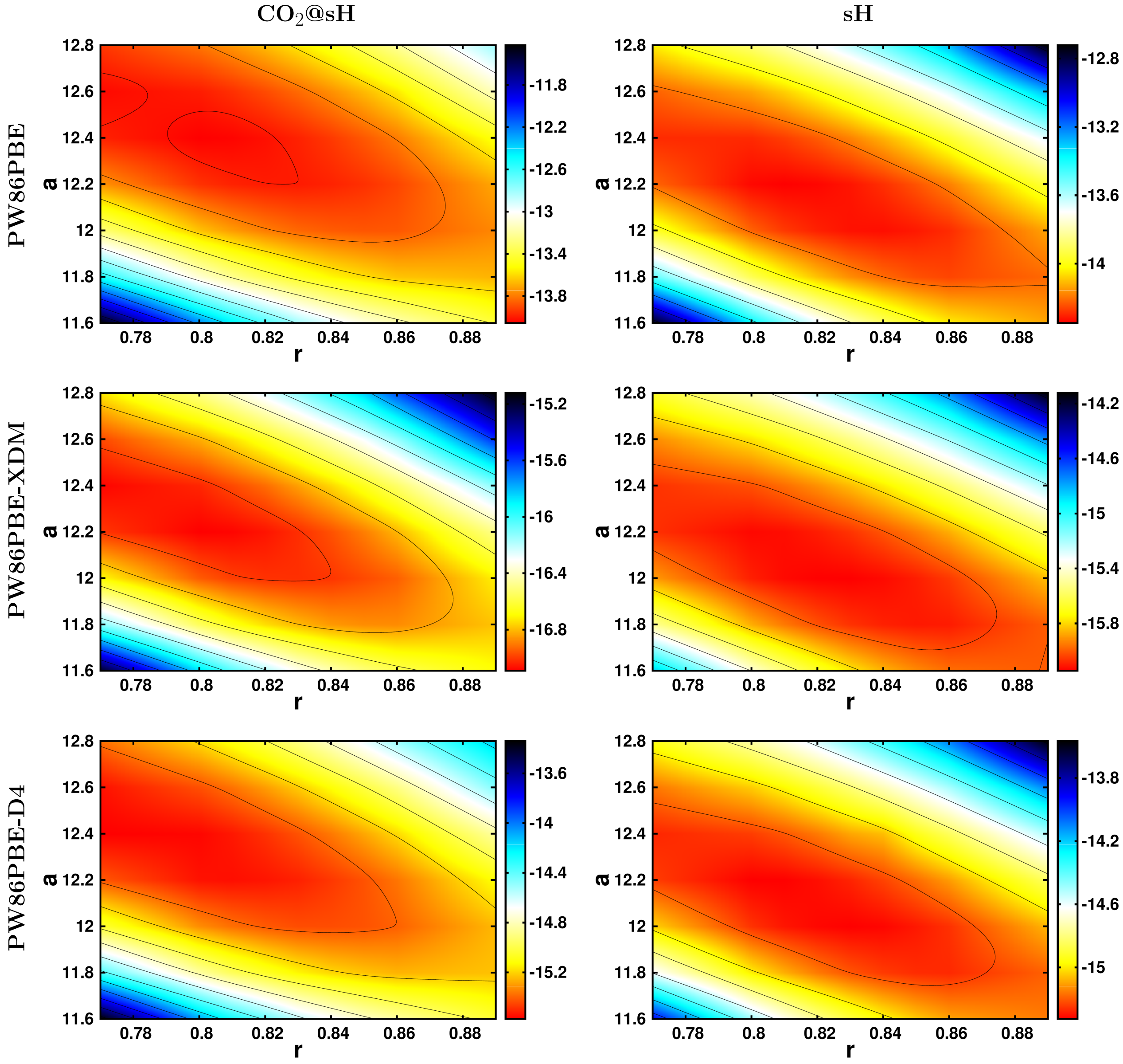

Table S2: CO_2 orientations in the empty sH and the fully filled CO_2@sH crystal unit cells as obtained from the PW86PBE-XDM geometry relaxation calculations. The six CO_2 molecules in the sH crystal unit cell are indicated with numbers (1-6) in Fig. 6.

Periodic unit cell (full optimization)					
Cage No.		θ (deg)	ϕ (deg)	r(Å)	Mean(Å)
D	1	89.828	4.420	0.156	0.189
	2	88.445	1.579	0.166	
	3	88.498	9.407	0.247	
D’	4	88.738	3.134	0.024	0.055
	5	89.272	0.734	0.087	
E	6	89.628	0.375	0.004	0.004
